# Pre-Exposure Prophylaxis–Related Messages Across Facebook, Instagram, and Twitter (Rebranded X): Content Analysis

**DOI:** 10.2196/87607

**Published:** 2026-09-30

**Authors:** Lourdes S Martinez, Nicole Crocker, Sharanya Akkone, Shravani Hariprasad, Rebecca Houghton, Eric R Walsh-Buhi

**Affiliations:** 1 School of Communication, San Diego State University, 5500 Campanile Drive, San Diego, CA, 92182, United States, 1 6572233122; 2 Department of Applied Health Science, School of Public Health, Indiana University Bloomington, Bloomington, IN, United States; 3 The Center for Human Dynamics in the Mobile Age, San Diego State University, San Diego, CA, United States

**Keywords:** social media, content analysis, pre-exposure prophylaxis, health communication, HIV

## Abstract

**Background:**

Interventions are needed to address the lack of pre-exposure prophylaxis (PrEP) awareness and mitigate barriers related to PrEP use. One such intervention modality is social media, as PrEP awareness and communication on issues, such as access and cost, are easily addressed via clear social media messages on platforms for PrEP-eligible people, and especially young people, who use social media more frequently.

**Objective:**

This descriptive study seeks to extend understanding of PrEP awareness and usage by examining PrEP-related communication across 3 popular social media platforms (Facebook [Meta Platforms, Inc], Instagram [Meta Platforms, Inc], and Twitter [rebranded as X]) and identifying message and source characteristics.

**Methods:**

In February 2023, we used CrowdTangle (a public insights tool owned by Facebook, now known as Meta) to gather a total of 39,790 Facebook posts and 5628 Instagram posts. We also used Twitter’s public API to collect 14,061 Twitter posts during the same time frame. Of these, we drew a random sample of social media posts from each platform (1000 posts from Facebook, 1000 posts from Instagram, and 811 posts from Twitter) in February 2023 and analyzed them using a quantitative content analysis.

**Results:**

Our findings showed some differences in the types of text-based content appearing on each platform. We also uncovered similar patterns across all three platforms: (1) definitions of and indications for PrEP were the most common type of text-based content in posts; (2) information about PrEP appearing in social media posts drew from websites other than traditional sources such as public health organizations, health care providers, and government agencies; and (3) men who have sex with men represented the most frequently mentioned target population. Although our study did not detect a large presence of theory-based concepts from behavior change theory, such as the integrative model of behavioral prediction, across all platforms, attitude emerged most frequently, followed by self-efficacy.

**Conclusions:**

These findings shed light on the PrEP-related content that may reach and shape the perceptions of young people who could benefit from PrEP use. Such insights can guide the design of future social media–based messages targeting the most influential beliefs to strengthen HIV prevention efforts.

## Introduction

HIV/AIDS remains a major public health problem in the United States [1]. Prevalence rates of HIV are unequally distributed [2,3], and health disparities for new HIV cases affect several communities, including those defined by race/ethnicity, sexual orientation, age, region, and injecting drug use (IDU) status [4]. In 2022, rates of newly diagnosed HIV infections were higher among men who have sex with men (MSM), specifically Black/African American and Hispanic/Latino MSM; Black/African American female individuals; Black/African American transgender persons; people aged 25 to 34 years; people residing in the southern region of the United States; and people who inject drugs [2,3].

Pre-exposure prophylaxis (PrEP) is a medication (with several names, including Truvada, Descovy, and Apretude) that can prevent HIV transmission among those exposed to HIV through sexual activity or injection drug use [1], but remains underused [5,6]. Although PrEP uptake has increased in recent years, the racial and gender gaps in PrEP use have only continued to widen [7,8]. To support future interventions needed to increase PrEP awareness, uptake, and adherence, the current study seeks to extend the understanding of PrEP awareness and usage by examining PrEP-related communication across 3 popular social media platforms (Facebook [Meta Platforms, Inc], Instagram [Meta Platforms, Inc], and Twitter [rebranded as X]) and identifying message and source characteristics. This descriptive study moves the literature forward beyond studies that focus solely on Twitter or Instagram and is novel in its ability to uncover intervention opportunities to address health disparities both within and across 3 social media platforms.

The proportions of PrEP awareness and use among MSM have increased since 2014, although awareness and use are still very low [9-14]. According to past research [15], while many at-risk individuals have heard of PrEP, their understanding of its use and benefits is often incomplete, particularly among racial minorities. According to a previous study, among 9359 total participants, 3026 (32.3%) were aware of PrEP, including 1221 (29.2%) men and 1805 (34.8%) women [16]. Among heterosexually active men, less than one-fifth (19.5%) of Hispanic participants, less than one-quarter (24.2%) of White participants, and less than one-third (31.9%) of Black participants were aware of PrEP. These results highlight an important gap in public awareness, particularly in Hispanic/Latino communities.

Interventions are needed to address the lack of PrEP awareness and mitigate barriers related to PrEP use. One such intervention modality is social media [17-19]. However, understanding of PrEP messaging on social media remains sparse. For many people, social media consumes a large amount of their day [20] and is an important source of news [21]. Research on social media use suggests as many as 70% of Americans aged 18 to 29 years use Facebook, 71% use Instagram, and 42% use Twitter [22]. Messages shared on social media platforms have the potential to impact public perceptions [23,24] and willingness to engage in health-protective behaviors [25], including a person’s willingness to use PrEP [26,27]. Priority populations for HIV prevention represent a large sum of social media users [28].

Studies in the extant literature have focused only on a few social media platforms, most notably Twitter (rebranded as X) [29-33]. Comparatively, much less is known about PrEP messaging and content on platforms other than Twitter, such as Instagram [34,35] and Facebook [33]. To our knowledge, no study has reviewed PrEP-related Facebook content, aside from evaluations of specific social media HIV prevention campaigns [36-39].

In this study, we applied behavior change theory [40] to uncover insights into key factors shaping PrEP behavior that may be shared on posts on social media platforms. The integrative model of behavioral prediction (IMBP) served as the guiding framework for the current study [40]. This theory is appropriate for our study as it points toward possible themes appearing in social media content that could prime or stimulate determinants of PrEP use and translate into offline PrEP behavior. According to the IMBP, behavior is predominantly driven by behavioral intention. Three factors constitute behavioral intention: attitudes, perceived norms, and self-efficacy. Each of the 3 factors shaping behavioral intention is, in turn, comprised of a set of behavioral beliefs salient to the performance of the behavior. We also applied the Multilevel Model of Meme Diffusion (M^3^D) [41,42] to identify features of posts that may help them spread across social media. Briefly, the M^3^D proposed that several factors accelerate the speed at which information proliferates over time. The M^3^D is relevant to this study by suggesting features of messages, users, and social networks that operate to diffuse information [43-46]. Given gaps in the extant literature, the present study aimed to identify PrEP messages that were more likely to be shared and to determine what variations existed across platforms. In addition, we examined theoretical constructs from the IMBP and M^3^D present in PrEP messages.

## Methods

### Ethical Considerations

Data for this study were gathered from publicly available sources. This study was approved by the institutional review board at the University of Indiana Bloomington (#26127) and was determined to be expedited. Any identifiable information has been removed from the presentation of our results, including names, usernames or handles, geographic locations, and other personal data.

### Data Collection

In February 2023, we used CrowdTangle (a public-insights tool owned by Facebook, now known as Meta) to gather a total of 39,790 publicly available Facebook posts and 5628 publicly available Instagram posts. We also used Twitter’s API to collect 14,061 publicly available Twitter posts during the same time period. Although we aimed to retrieve all relevant posts from February 2, 2022 midnight to February 2, 2023 11:59 PM, new Twitter ownership and API policies at the time of data collection restricted us to posts from December 12, 2022, to February 2, 2023. A description of CrowdTangle [47] as a tool for gathering social media posts is available elsewhere [35]. For all 3 social media platforms, we used the following predefined keywords, based on prior research [35] with some additions for the current research, to identify PrEP-related posts originating from users in the United States: “Truvada,” “Emtricitabine,” “Tenofovir,” “pre-exposure prophylaxis,” “#truvada,” “#truvadaprep,” “Descovy,” “#descovy,” “cabotegravir,” “Apretude,” and “(PrEP HIV), (PrEP AIDS).” Sample sizes of 1000 posts have been used in prior content analyses of Instagram [48], Facebook, and Twitter [49]. The sample size of 1000 posts also allowed us to balance different study needs concerning feasibility in random sampling, manual coding, and analytic goals that required avoiding violations of mutual exclusivity while preserving comparability across platforms. This approach considered differences across platforms in content redundancy we expected during deduplication efforts (especially with Twitter) based on past research [50,51], while achieving sufficiently large and comparable sample sizes from each platform to detect even small statistical differences within and across platforms. To generate our analytical sample for each platform, we randomly sampled 1000 unique social media posts from unique users to ensure each social media post and user appeared only once in each analytical sample. Inclusion criteria for our analytical samples required that social media posts were written in English and were specifically relevant to PrEP. Although our final analytical samples for Facebook and Instagram remained at 1000 cases, our final analytical sample for Twitter was reduced to 811 cases due to redundancies in the data.

### Coding Procedures

We used a codebook to guide a quantitative content analysis of social media posts. All data were manually coded, and our codebook contained categories, definitions, examples, and procedures for manually coding social media posts adapted from prior work. Specifically, our codebook draws from past research guiding the coding process for source attribution [52], source characteristics [50,51], and information about PrEP, including visual characteristics of posts [53-55], use characteristics of PrEP [56,57], stigma [29], and intended target audiences based on race [58], gender [59,60], and risk factors [59]. We also used theory-driven elements guided by the M^3^D [41,42] to capture the presence of concepts predictive of the memes’ viral behavior online and concepts from the IMBP [40] to gather evidence for determinants of human users’ PrEP-related behavior offline. We developed measures to code for other genders, IDU, other sources of information not included in existing measures (eg, information shared by celebrities, health departments, the World Health Organization, and other web sources), and characteristics of personal stories about PrEP use.

Variables for each platform were manually coded separately using codebooks slightly modified to accommodate the distinct properties of each platform. We first developed a codebook and established intercoder reliability for the Facebook sample, given that Facebook posts contained the most text in our data. This codebook provided the baseline procedures that were later adapted for the Instagram and Twitter codebooks. Prior to manually coding the Facebook sample, we completed a reliability analysis. During this reliability analysis, 2 coders independently coded a random subset of 100 posts (representing 10% of the analytical sample) and convened to discuss and resolve coding differences. Coders achieved good intercoder reliability for all variables after 4 rounds of coding (average Gwet [61] agreement coefficient=0.94, ranging from 0.80 to 0.99). Posts not included in the reliability analysis were single-coded after coders reached good intercoder reliability across all variables in the codebook. Once coding for the Facebook sample was completed, we repeated this process for the Instagram and Twitter samples, respectively. For the Instagram sample, coders achieved good intercoder reliability for all variables after 2 rounds of coding (average Gwet agreement coefficient=0.95, ranging from 0.80 to 0.99), and after 2 rounds of coding (average Gwet agreement coefficient=0.96, ranging from 0.83 to 0.99) for the Twitter sample. We used Gwet [61] agreement coefficient to calculate intercoder reliability, given its advantages in overcoming sensitivity to prevalence that may limit other intercoder reliability statistics such as Cohen κ and Scott π [62,63]. Intercoder reliability was calculated using the software AgreeStat (version 2015.6; Advanced Analytics, LLC).

### Data Analysis

To provide a description of our sample’s characteristics, we performed a series of frequency distributions displayed in Tables 1-4, including intended target audiences of PrEP messages (Table 1), sources attributed to PrEP information appearing in posts and characteristics of personal experiences or accounts regarding PrEP (Table 2), source characteristics of PrEP messages (Table 3), and social media users’ engagement with PrEP messages (Table 4; available only for Facebook and Instagram). For Table 1, Twitter posts were not coded for visual-based content due to the properties of the platform. Table 4 displays engagement metrics after min-mix normalization; however, some engagement metrics were not included for Twitter and Instagram due to the unavailability of certain data from these platforms. For example, given that sharing metrics were available for Facebook but not Instagram, no data were missed when discussing platform engagement differences. Similarly, some M^3^D concepts were not coded for Twitter and Instagram due to a lack of feasibility in our manual coding process. As detailed results are provided in these tables, we summarize key findings here. To address our first study objective, we performed a series of cross-tabular analyses to test for significant associations between variables of interest while reporting effect sizes. We also used frequency distributions to achieve our second study objective. Data were analyzed using the software SPSS Statistics (version 30.0; IBM).

**Table 1.** Content characteristics: information about pre-exposure prophylaxis (PrEP) mentioned in posts^a^.

Characteristics	Facebook (n=1000), n (%)	Instagram (n=1000), n (%)	Twitter (rebranded as X; n=811), n (%)
Text content			
PrEP defined	327 (32.7)	341 (34.1)	95 (11.7)
How PrEP works	12 (1.2)	9 (0.9)	4 (0.5)
Who can use PrEP	167 (16.7)	162 (16.2)	92 (11.3)
Effectiveness	100 (10)	90 (9)	45 (5.5)
How to get PrEP	195 (19.5)	166 (16.6)	35 (4.3)
Costs	77 (7.7)	129 (12.9)	58 (7.2)
Side effects	12 (1.2)	5 (0.5)	22 (2.7)
Promoting use of PrEP	155 (15.5)	101 (10.1)	60 (7.4)
Stigma	10 (1)	10 (1)	16 (1.9)
Antistigma	11 (1.1)	8 (0.8)	2 (0.2)
Race	60 (6)	116 (11.6)	15 (1.8)
Male	66 (6.6)	128 (12.8)	63 (7.8)
Female	75 (7.5)	106 (10.6)	25 (3.1)
Other gender	19 (1.9)	68 (6.8)	4 (0.5)
Transgender	98 (9.8)	238 (23.8)	26 (3.2)
Heterosexual	10 (1)	20 (2)	20 (2.5)
IDU^b^	17 (1.7)	21 (2.1)	5 (0.6)
MSM^c^	137 (13.7)	302 (30.2)	89 (10.9)
Visual content			
Photo	824 (82.4)	872 (87.2)	—^d^
Infographic	122 (12.2)	120 (12)	—
Video	72 (7.2)	105 (10.5)	—
One person depicted	157 (15.7)	249 (24.9)	—
Two people depicted	85 (8.5)	87 (8.7)	—
Three or more people depicted	124 (12.4)	159 (15.9)	—

a Each line represents the number and percentage of cases in which a specific element was detected. The numbers and percentages of cases in which a specific characteristic was not detected are not displayed.

b IDU: injecting drug use.

c MSM: men who have sex with men.

d Not applicable.

**Table 2.** Content characteristics: information about pre-exposure prophylaxis (PrEP) source attribution and personal accounts^a^.

Characteristics	Facebook (n=1000), n (%)	Instagram (n=1000), n (%)	Twitter (rebranded as X; n=811), n (%)
Source attribution			
CDC^b^	31 (3.1)	29 (2.9)	5 (0.6)
Medical doctors	37 (3.7)	20 (2)	11 (1.4)
Government officials	32 (3.2)	20 (2)	8 (0.9)
Gilead (manufacturer of PrEP)	4 (0.4)	6 (0.6)	13 (1.6)
Cancer organizations	1 (0.1)	1 (0.1)	0 (0)
Members of research community	22 (2.2)	50 (5)	31 (3.8)
Other web source (not included above)	110 (11)	125 (12.5)	60 (7.4)
Celebrity	0 (0)	8 (0.8)	0 (0)
Health department (state or local)	22 (2.2)	14 (1.4)	24 (2.9)
WHO^c^	5 (0.5)	7 (0.7)	4 (0.5)
Personal accounts			
Personal firsthand experience with PrEP	11 (1.1)	8 (0.8)	46 (5.7)
Age when personal account occurred	—^d^	—	—
Youth (under 18)	3 (0.3)	0 (0)	0 (0)
Adult (18 and older)	0 (0)	0 (0)	0 (0)
Personal account from MSM^e^	1 (0.1)	0 (0)	4 (0.5)

a Each line represents the number and percentage of cases in which a specific element was detected. The numbers and percentages of cases in which a specific characteristic was not detected are not displayed.

b CDC: Centers for Disease Control and Prevention.

c WHO: World Health Organization.

d Not applicable.

e MSM: men who have sex with men.

**Table 3.** Source characteristics^a^.

Characteristics	Facebook (n=1000), n (%)	Instagram (n=1000), n (%)	Twitter (rebranded as X; n=811), n (%)
Organization	943 (94.3)	704 (70.4)	182 (22.4)
Business	418 (41.8)	313 (31.3)	42 (5.2)
Nonprofit	403 (40.3)	215 (21.5)	70 (8.6)
Government	93 (9.3)	85 (8.5)	21 (2.6)
News media	154 (15.4)	70 (7)	16 (1.9)
School	24 (2.4)	14 (1.4)	8 (0.9)
Health information provider	263 (26.3)	111 (11.1)	53 (6.5)
Health care organization	184 (18.4)	149 (14.9)	38 (4.7)
Nonhealth advocacy group	113 (11.3)	91 (9.1)	13 (1.6)
Individual	57 (5.7)	296 (29.6)	628 (77.4)
Mother, father, or parent	0 (0)	22 (2.2)	9 (1.1)
Son, daughter, or child	1 (0.1)	1 (0.1)	0 (0)
Spirituality	1 (0.1)	2 (0.2)	17 (2.1)
Political persuasion	22 (2.2)	49 (4.9)	6 (0.7)
Journalist	1 (0.1)	10 (1)	13 (1.6)
Physician	0 (0)	19 (1.9)	28 (3.5)
Epidemiologist	1 (0.1)	0 (0)	1 (0.1)
Health educator	1 (0.1)	15 (1.5)	5 (0.6)
Nurse other health worker	3 (0.3)	8 (0.8)	43 (5.3)

a Each line represents the number and percentage of cases in which a specific element was detected. The numbers and percentages of cases in which a specific characteristic was not detected are not displayed.

**Table 4.** Public reactions to content and theory-based elements^a^.

Characteristics	Facebook (n=1000)		Instagram (n=1000)		Twitter (rebranded as X; n=811)	
Reactions to content, mean (SD)						
Likes	0.008 (0.06)		0.004 (0.04)		—^b^	
Comments	0.003 (0.04)		0.011 (0.06)		—	
Shares	0.007 (0.05)		—		—	
M^3^D^c^ concepts						
Popularity, mean (SD)	5.8 (40.4)		—		—	
Trialability, n (%)	60 (6)		19 (1.9)		96 (11.8)	
Distinctiveness, n (%)	66 (6.6)		22 (2.2)		8 (0.9)	
Credibility	—		—		—	
Lay person, n (%)	788 (78.8)		873 (87.3)		—	
Expert, n (%)	212 (21.2)		127 (12.7)		—	
Motivation, n (%)	37 (3.7)		4 (0.4)		1 (0.1)	
Network size, mean (SD)	0.007 (0.05)		0.009 (0.05)		—	
IMBP^d^ concepts, n (%)						
Intention	8 (0.8)		13 (1.3)		36 (4.4)	
Attitude	289 (28.9)		288 (28.8)		191 (23.6)	
Injunctive norms	1 (0.1)		1 (0.1)		0 (0)	
Descriptive norms	0 (0)		0 (0)		0 (0)	
Self-efficacy	99 (9.9)		159 (15.9)		87 (10.7)	

a Each line represents the number and percentage of cases in which a specific element was detected. The numbers and percentages of cases in which a specific characteristic was not detected are not displayed.

b Not applicable.

c M3D: Multilevel Model of Meme Diffusion.

d IMBP: integrative model of behavioral prediction.

## Results

### Source Characteristics and PrEP-Related Information by Platform

We first identified source characteristics of posts, which showed that the majority of sources of posts on Facebook and Instagram were accounts run by organizations, whereas the opposite was true for Twitter, where most sources represented individual user accounts. For both Facebook and Instagram, the most common categories for organizations were businesses and nonprofits and, to a lesser extent, health information providers and nonhealth advocacy groups. For accounts representing individual users on Twitter, the most frequently observed categories were physicians and nurses/other health care workers. For each type of PrEP-related information available in text or visual formats, we then examined which types of organizations were more likely to feature that content. Below, we report statistically significant results for each platform. All other associations between types of PrEP-related information and account type were not statistically significant.

### PrEP-Related Information in Posts From Different Account Types on Facebook

On Facebook, we found that nonprofits were statistically more likely to share posts mentioning race (φ=0.10, *P*=.001), transgender (φ=0.11, *P*<.001), and MSM (φ=0.13, *P*<.001), compared to organizations that did not fall under this category. Similarly, nonhealth advocacy groups were also statistically more likely to share posts mentioning transgender (φ=.20, *P*<.001) and MSM (φ=0.15, *P*<.001), but less likely to include definitions of PrEP (φ=–0.07, *P*=.03). While government agencies were also statistically more likely to share posts including content promoting PrEP use (φ=0.09, *P*=.004), definitions of PrEP (φ=0.07, *P*=.03), and ways to get PrEP (φ=0.07, *P*=.03), they were statistically less likely than organizations not designated under this category to mention transgender (φ=–0.09, *P*=.003) and MSM (φ=–0.12, *P*<.001). Although news media were more likely to mention who can use PrEP (φ=0.06, *P*=.05), these organizations were also less likely to offer content promoting PrEP use (φ=–0.08, *P*=.01). In contrast, organizations representing health information providers were statistically more likely to promote PrEP use (φ=0.08, *P*=.01) but less likely to mention transgender (φ=–0.07, *P*=.03). Finally, compared to other organization categories, health care organizations discussed PrEP effectiveness (φ=0.07, *P*=.04), ways to get PrEP (φ=0.16, *P*<.001), and promoted PrEP (φ=0.14, *P*<.001). No other associations between organization types and content (text or visual) were observed, nor did we observe statistically significant differences in content posted from accounts operated by individuals and organizations. The overall pattern of φ coefficients showed small effect sizes (φ<0.3), suggesting weak associations between organization types and their posted PrEP-related content on Facebook.

### PrEP-Related Information in Posts From Different Account Types on Instagram

Unlike on Facebook, we observed differences in content shared by accounts operated by individuals and organizations. For example, organizations were statistically significantly more likely to include content about PrEP effectiveness (φ=0.07, *P*=.04) and IDU (φ=0.08, *P*=.01), while accounts operated by individuals were statistically more likely to mention costs (φ=–0.15, *P*<.001) and heterosexual individuals (φ=–0.11, *P*<.001) in their text-based content. For visual-based content, organizations were statistically significantly more likely than accounts operated by individuals to include a photo (φ=0.09, *P*=.02), infographic (φ=0.11, *P*=.003), video (φ=0.10, *P*=.004), and depictions of 1 person (φ=0.23, *P*<.001) or 3 or more people (φ=0.10, *P*=.02).

In terms of content shared by different types of organizations, we observed that businesses were statistically significantly more likely to provide content that included definitions for PrEP (φ=0.07, *P*=.02) and visual-based content depicting an individual (φ=0.09, *P*=.02), but were less likely to actively promote PrEP usage (φ=–0.08, *P*=.02). In addition to avoiding references to stigma (φ=–0.06, *P*=.05), news media were similarly likely to offer definitions of PrEP (φ=0.12, *P*<.001) and avoid actively promoting PrEP usage (φ=–0.08, *P*=.01); however, these organizations were also statistically more likely to include content about who can use PrEP (φ=0.10, *P*<.001) and PrEP effectiveness (φ=0.09, *P*=.004). Organizations classified as government agencies were statistically less likely to include content referencing race (φ=–0.08, *P*=.02), transgender (φ=–0.10, *P*=.003), other genders (φ=–0.08, *P*=.009), and MSM (φ=–0.12, *P*<.001). In addition to avoiding mentions of costs (φ=0.23, *P*<.001), health information providers were also statistically less likely to include references to transgender (φ=0.23, *P*<.001), other genders (φ=0.23, *P*<.001), and MSM (φ=0.23, *P*<.001). Health care organizations similarly avoided mentioning race (φ=–0.07, *P*=.02), transgender (φ=–0.06, *P*=.05), and MSM (φ=–0.09, *P*=.004), but were more likely to include content defining PrEP (φ=0.08, *P*=.008) and ways to get PrEP (φ=0.08, *P*=.01). Although nonhealth advocacy groups were more likely to share content mentioning race (φ=0.10, *P*<.001), transgender (φ=0.09, *P*=.003), and MSM (φ=0.09, *P*=.006), they were less likely to post content defining PrEP (φ=–0.06, *P*=.04). Lastly, nonprofits were statistically more likely to reference race (φ=0.07, *P*=.02) and transgender (φ=0.06, *P*=.04) in their text-based content, as well as include depictions of an individual in their visual-based content (φ=0.08, *P*=.03), but were less likely to mention costs (φ=–0.09, *P*=.007) and heterosexual individuals (φ=–0.08, *P*=.009). As with Facebook, the overall pattern of φ coefficients demonstrated small effect sizes (φ<0.3), indicating weak associations between organization types and posted PrEP-related content on Instagram.

### PrEP-Related Information in Posts From Different Account Types on Twitter

We only observed a few differences in text-based, PrEP-related information shared in posts from different account types on Twitter. Of the differences we noted, organizations were statistically significantly more likely to share information defining PrEP (φ=0.12, *P*<.001), who can use PrEP (φ=0.13, *P*<.001), PrEP effectiveness (φ=0.13, *P*<.001), and how to get PrEP (φ=0.09, *P*=.009). Looking more closely at content shared by different types of organizations, we observed that health information providers were also more likely to share content that defined PrEP (φ=0.12, *P*<.001). Like Facebook and Instagram, we saw an overall pattern of φ coefficients with small effect sizes (φ<0.3), denoting weak associations between organization types and posted PrEP-related content on Twitter.

We also found statistically significant differences across platforms for most of the text-based content displayed in Table 5. Information included in posts shared on Twitter was least likely to define PrEP (*χ*²_2_=135.9, *P*<.001, Cramer *V*=0.22) and least likely to mention who could use PrEP (*χ*²_2_=11.6, *P*=.003, Cramer *V*=0.07). Twitter posts were also least likely to provide information regarding the effectiveness of PrEP (*χ*²_2_=12.6, *P*=.002, Cramer *V*=0.07) and how to obtain PrEP (*χ*²_2_=97.5, *P*<.001, Cramer *V*=0.19). Compared to other platforms, Twitter was also most likely to mention PrEP side-effects (*χ*²_2_=16.2, *P*<.001, Cramer *V*=0.08) and least likely to mention IDU (*χ*²_2_=6.7, *P*=.04, Cramer *V*=0.05).

**Table 5.** Content characteristics: information about pre-exposure prophylaxis (PrEP) mentioned in posts across different platforms (N=2712)^a^.

Characteristics	Facebook, n (%)	Instagram, n (%)	Twitter (rebranded as X), n (%)	Chi-square (*df*)	*P* value	Cramer *V*
Text content						
PrEP defined				135.9 (2)	<.001	0.22
No	636 (66.8)	637 (66.5)	709 (88.4)			
Yes	316 (33.2)	321 (33.5)	93 (11.6)			
How PrEP works				2.2 (2)	.33	0.028
No	941 (98.8)	949 (99.1)	798 (99.5)			
Yes	11 (1.2)	9 (0.9)	4 (0.5)			
Who can use PrEP				11.6 (2)	.003	0.065
No	792 (83.2)	805 (84.0)	711 (88.7)			
Yes	160 (16.8)	153 (15.9)	91 (11.3)			
Effectiveness				12.6 (2)	.002	0.068
No	856 (89.9)	875 (91.3)	758 (94.5)			
Yes	96 (10.1)	83 (8.7)	44 (5.5)			
How to get PrEP				97.5 (2)	<.001	0.19
No	763 (80.1)	798 (83.3)	769 (95.9)			
Yes	189 (19.9)	160 (16.7)	33 (4.1)			
Costs				22.5 (2)	<.001	0.091
No	877 (92.1)	832 (86.8)	744 (92.8)			
Yes	75 (7.9)	126 (13.2)	58 (7.2)			
Side effects				16.2 (2)	<.001	0.077
No	941 (98.8)	953 (99.5)	780 (97.3)			
Yes	11 (1.2)	5 (0.5)	22 (2.7)			
Promoting use of PrEP				30.7 (2)	<.001	0.106
No	807 (84.8)	867 (90.5)	743 (92.6)			
Yes	145 (15.2)	91 (9.5)	59 (7.4)			
Stigma				3.8 (2)	.14	0.038
No	942 (98.9)	948 (98.9)	786 (98.0)			
Yes	10 (1.1)	10 (1.0)	16 (1.9)			
Antistigma				4.9 (2)	.09	0.042
No	941 (98.8)	951 (99.3)	800 (99.8)			
Yes	11 (1.2)	7 (0.7)	2 (0.2)			
Race				71.8 (2)	<.001	0.163
No	897 (94.2)	844 (88.1)	787 (98.1)			
Yes	55 (5.8)	114 (11.9)	15 (1.9)			
Male				25.1 (2)	<.001	0.096
No	892 (93.7)	837 (87.4)	739 (92.1)			
Yes	60 (6.3)	121 (12.6)	63 (7.9)			
Female				35.7 (2)	<.001	0.115
No	880 (92.4)	857 (89.5)	777 (96.9)			
Yes	72 (7.6)	101 (10.5)	25 (3.1)			
Other gender				62.2 (2)	<.001	0.151
No	933 (98.0)	893 (93.2)	798 (99.5)			
Yes	19 (2.0)	65 (6.8)	4 (0.5)			
Transsexual				182 (2)	<.001	0.259
No	859 (90.2)	727 (75.9)	776 (96.8)			
Yes	93 (9.8)	231 (24.1)	26 (3.2)			
Heterosexual				6.5 (2)	.04	0.049
No	943 (99.1)	938 (97.9)	782 (97.5)			
Yes	9 (0.9)	20 (2.1)	20 (2.5)			
IDU^b^				6.7 (2)	.04	0.05
No	935 (98.2)	938 (97.9)	797 (99.4)			
Yes	17 (1.8)	20 (2.1)	5 (0.6)			
MSM^c^				134.8 (2)	<.001	0.223
No	824 (86.6)	666 (69.5)	713 (88.9)			
Yes	128 (13.4)	292 (30.5)	89 (11.1)			
Visual content						
Photo						
No	158 (16.8)	101 (10.8)	N/A^d^	—^e^	—	—
Yes	783 (83.2)	832 (89.2)	N/A	—	—	—
Infographic						
No	826 (87.8)	817 (87.6)	N/A	—	—	—
Yes	115 (12.2)	116 (12.4)	N/A	—	—	—
Video						
No	872 (92.7)	830 (88.9)	N/A	—	—	—
Yes	69 (7.3)	103 (11.0)	N/A	—	—	—
One person depicted						
No	793 (84.0)	693 (74.3)	N/A	—	—	—
Yes	151 (16.0)	240 (25.7)	N/A	—	—	—
Two people depicted						
No	861 (91.2)	849 (90.9)	N/A	—	—	—
Yes	83 (8.8)	84 (9.0)	N/A	—	—	—
Three or more people depicted						
No	826 (87.5)	777 (83.3)	N/A	—	—	—
Yes	118 (12.5)	156 (16.7)	N/A	—	—	—

a Cases for visual variables of Facebook and Instagram are less than 1000 due to some cases not having any visual content available.

b IDU: injecting drug use.

c MSM: men who have sex with men.

d Not available.

e Not applicable.

For information shared on Instagram, posts made on this platform were most likely to mention costs associated with PrEP use (*χ*²_2_=22.5, *P*<.001, Cramer *V*=0.09) and race (*χ*²_2_=71.8, *P*<.001, Cramer *V*=0.16). Instagram posts were also most likely to include information about male gender (*χ*²_2_=25.1, *P*<.001, Cramer *V*=0.10), female gender (*χ*²_2_=35.7, *P*<.001, Cramer *V*=0.12), and other genders (*χ*²_2_=62.2, *P*<.001, Cramer *V*=0.15). Compared to Facebook and Twitter, Instagram posts were also most likely to offer information for transgender individuals (*χ*²_2_=182, *P*<.001, Cramer *V*=0.26) and MSM (*χ*²_2_=134.8, *P*<.001, Cramer *V*=0.22). In contrast, when compared to both Instagram and Twitter, posts shared on Facebook were most likely to include information promoting PrEP (*χ*²_2_=30.7, *P*<.001, Cramer *V*=0.11) and least likely to mention heterosexual individuals (*χ*²_2_=6.5, *P*=.047, Cramer *V*=0.05). All observed differences resulted in an overall pattern of small effect sizes, with Cramer *V* values below 0.3, signifying weak associations between messages featured on different platforms and the types of target populations or audiences intended for those messages.

We also examined the presence of IMBP and M^3^D theory–based elements, the results of which are provided in Table 4. Regarding the presence of IMBP elements, attitude (Facebook: 289/1000, 28.9%; Instagram: 288/1000, 28.8%; Twitter: 191/811, 23.6%) appeared with the greatest frequency, followed by self-efficacy (Facebook: 99/1000, 9.9%; Instagram: 159/1000, 15.9%; Twitter: 87/811, 10.7%) across all platforms. Intention was mentioned at a lower frequency on every platform (Facebook: 8/1000, 0.8%; Instagram: 13/1000, 1.3%; Twitter: 36/811, 4.4%). For all 3 platforms, injunctive norms (1/1000, 0.1%) almost never appeared and descriptive norms (0%) were never mentioned. For M^3^D elements, we observed few instances of trialability (Facebook: 60/1000, 6%; Instagram: 19/1000, 1.9%; Twitter: 96/811, 11.8%), distinctiveness (Facebook: 66/1000, 6.6%; Instagram: 22/1000, 2.2%; Twitter: 8/811, 0.9%), and motivation (Facebook: 37/1000, 3.7%; Instagram: 4/1000, 4%; Twitter: 1/811, 0.1%) across all 3 platforms. User credibility as a lay person (Facebook: 788/1000, 78.8%; Instagram: 873/1000, 87.3%) and user expertise (Facebook: 212/1000, 21.2%; Instagram: 127/1000, 12.7%) appeared with high frequency on Facebook and Instagram, both of which also had users with social network sizes greater than 100,000. However, we were unable to assess the presence of these elements in Twitter due to a lack of available data on users. Similarly, we were only able to assess user popularity for similar reasons.

## Discussion

### Principal Findings

This study examined which PrEP messages were more likely to be shared by which source types, differences in PrEP messages across platforms regarding their intended audiences, and the presence of theory-based elements in PrEP-related posts. Our study showed that many information sources mentioned in posts across all platforms tended to be sources that were not included in our codebook. This suggests that information about PrEP appearing in social media posts may not draw from sources such as public health agencies, medical physicians, and government officials, or others one might expect. Similarly, prior research found that only about 8% of posts analyzed in that study came from a health professional and 15% came from a health care organization [32]. In determining which content characteristics were more likely to be present in posts from different account types on each platform, our results showed that, while some types of organizations were statistically significantly more likely to share posts mentioning specific priority populations and PrEP-related content, others were not. These findings aligned with existing research [64], though some nuances suggested divergence [38]. When comparing PrEP content across platforms, results showed differences in intended audiences for most text-based content. LGBTQ young people have reported higher rates of feeling safe and understood on Instagram, compared to Twitter and Facebook, which may have explained why information on PrEP use for sexual and gender minorities was more prevalent on Instagram [65]. Results detecting only a small presence of theoretical constructs converge with prior research suggesting opportunities to increase use of behavior change theory in interventions [66,67]. As behavior change theory can guide interventions in audience analysis and message design [40], PrEP-related messages delivered on social media should aim to integrate the use of theory in future interventions [67]. Similarly, we only observed a minimal presence of M^3^D elements related to trialability, distinctiveness, and motivation across all 3 platforms. However, user credibility and user expertise appeared with notable frequency for Facebook and Instagram. Given concerns surrounding health misinformation and its implications for public health, the importance of user credibility and expertise may have represented one way to attract greater public engagement with quality health information on social media [68].

Implications of our study point toward several opportunities for intervention to help reduce or eliminate existing racial and gender health disparities. The use of nontraditional sources being prominent when designing effective future messages may help establish credibility or expertise for users seeking PrEP information, especially if their informational needs are not being met by information sources where they expect to find them. In line with this, our results suggest a number of content areas that may be lacking for a range of organizations engaged in PrEP-related communication on social media. We also note intervention opportunities for organizations that may have accounts across platforms to improve their public health communication strategies and awareness efforts, ensuring greater alignment across platforms. Lastly, including content designed to increase pro-PrEP social norms and self-efficacy may increase PrEP intentions that ultimately increase rates of PrEP usage [69]. To attract and maintain attention [41,42], PrEP-related content posted across all platforms could improve content distinctiveness by emphasizing new information or perspectives in the form of breaking news or novel scientific discoveries around PrEP. To facilitate ease of sharing [41,42], all platforms in our study have opportunities for increasing traction by enhancing the trialability of PrEP-related content, shortening and simplifying messaging around PrEP without sacrificing informational accuracy.

### Limitations and Directions for Future Research

Our study had limitations worthy of discussion. Although we were successful in operationalizing all theoretical concepts from the IMBP, we struggled to execute operationalizations for some theoretical concepts in the M^3^D due to a lack of data availability from the platforms, which limited our ability to fully compare the 3 platforms. Other operationalizations were excluded from our study due to feasibility. Thresholds included in our coding scheme were established based on properties of the platform(eg, Facebook limits clip length to 2 min) and/or industry recommendations for increasing user engagement (eg, best character length for posts based on the platform), further limiting our study.

Another limitation was that this study was largely descriptive and frequency-based in nature, and focused on the presence or absence of coded theoretical constructs. We acknowledged that there was no demonstration that these frameworks meaningfully structured interpretation or generated testable insights. Our study did not formally test these models or explanatory linkages; rather, it provided descriptive mapping of theoretical elements. These areas may offer fertile areas for future research.

While our use of Facebook, Twitter, and Instagram for content analysis provided valuable insights, our study was also limited by the selection of these particular platforms. They represented only a portion of the online discourse, potentially overlooking important conversations occurring on other social media platforms like YouTube (Alphabet, Inc) and TikTok (ByteDance), which are less text-focused than the platforms we analyzed. Moreover, our content analysis inherently focused on publicly shared content, which may not have fully captured the genuine feelings or thoughts of individuals who simply encounter or read this content without engaging with it. This approach primarily reflected what people choose to post, rather than their underlying beliefs or private reflections. It also did not include an analysis of comments or replies to the posts included in our study. Future research could expand on our study by incorporating additional social media platforms such as YouTube and integrating a mixed methods approach, such as combining content analysis with surveys or interviews to offer deeper insight into both expressed and unexpressed public sentiments, providing a deeper understanding of the impact and perception of PrEP across different segments of the population.

These findings helped shed light on the PrEP-related beliefs shaping young people’s perceptions and engagement. Such insights can guide the design of future social media–based messages, targeting the most influential beliefs to strengthen HIV prevention efforts. In an era marked by significant cuts to HIV prevention funding (including vaccine research [69] and global initiatives [70]), translating this knowledge into action is not optional; it is essential to controlling the HIV epidemic.

## Abbreviations

IDU: injecting drug use
IMBP: integrative model of behavioral prediction
M^3^D: Multilevel Model of Meme Diffusion
MSM: men who have sex with men
PrEP: pre-exposure prophylaxis
